# A cognitively demanding working-memory intervention enhances extinction

**DOI:** 10.1038/s41598-020-63811-0

**Published:** 2020-04-27

**Authors:** Lycia D. de Voogd, Elizabeth A. Phelps

**Affiliations:** 1 Department of Psychology, New York University, New York, NY 10003 USA; 2 Department of Psychology, Harvard University, Cambridge, MA 02138 USA

**Keywords:** Amygdala, Human behaviour

## Abstract

Improving extinction learning has the potential to optimize psychotherapy for persistent anxiety-related disorders. Recent findings show that extinction learning can be improved with a cognitively demanding eye-movement intervention. It is, however, unclear whether [1] any cognitively-demanding task can enhance extinction, or whether it is limited to eye movements, and [2] the effectiveness of such an intervention can be enhanced by increasing cognitive load. Participants (n = 102, n = 75 included in the final sample) completed a Pavlovian threat conditioning paradigm across two days. One group underwent standard extinction (Control), a second group underwent extinction paired with a 1-back working memory task (Low-Load), and a third group underwent extinction paired with a 2-back working memory task (High-Load). We found that the conditioned response during extinction was reduced for both the Low-Load and the High-Load groups compared to the Control group. This reduction persisted during recovery the following day when no working memory task was executed. Finally, we found that within the High-Load group, participants with lower accuracy scores on the 2-back task (i.e., for who the task was more difficult) had a stronger reduction in the conditioned response. We did not observe this relationship within the Low-Load group. Our findings suggest that cognitive load induced by a working memory intervention embedded during extinction reduces persistent threat responses.

## Introduction

Extinction learning is the process through which a learned threat response is no longer expressed after the repeated presentation of a previously threatening stimulus^1^. This process of extinction learning is a core feature of most effective therapies for disorders of fear and anxiety, such as exposure therapy^2^. Through repeated exposure, patients experience a reduction in fear and anxiety-related symptoms. Although exposure can be initially successful in reducing symptoms, these symptoms can often return^3^. Therefore, improving extinction learning has the potential to optimize treatment for persistent fear and anxiety-related disorders.

One way extinction learning may be improved is through an eye movement intervention^4^. This idea is based on the notion that goal-directed eye movements form an important part of eye movement desensitization and reprocessing (EMDR). Like exposure therapy, EMDR is also an evidence-based therapy for fear and anxiety-related disorders^2^. During EMDR treatment, patients alternate their attention between recalling traumatic memories and making lateral eye movements. Eye movements are central to EMDR, but there has been a debate as to whether they play a role in the therapeutic outcome above standard extinction^5,6^. A meta-analysis comparing EMDR treatment with and without eye movements indicated, however, that eye movements have an added value to the treatment^7^. Moreover, it was recently found that persistent threat responses were reduced when goal-directed eye movements were embedded during extinction learning, compared to standard extinction when no additional task was executed^4^, suggesting a benefit of eye movements above standard extinction. The notion that the execution of goal-directed eye movements during extinction learning can reduce persistent threat responses has also recently been found in animal models^8^.

How does EMDR treatment work? It has been suggested that eye movements tax working memory and thereby compete with recalling traumatic memories^9^ making the memory less vivid and emotional^9,10^. In line with this, experimental studies have shown that the execution eye movements or other cognitively demanding tasks together with recalling emotional memories indeed reduce the vividness and emotionality of such memories^11^. Moreover, it has been shown that the effectiveness of such tasks are dependent on the difficulty^11–13^. These findings form the basis of a cognitive working model of EMDR^9,10,14^. A crucial assumption of this model is that because both eye movements and recalling traumatic memories tax working memory they interfere with each other. We propose an extension to this model^15^ and suggest that a cognitively demanding task by itself can interfere with threat-related processes even when the the threat-related task imposes minimal working memory demands. In support of this hypothesis, it has been shown that during working memory maintenance, threat conditioning is impaired^16^ and threat-potentiated startle responses are decreased^17^.

A potential neurobiological model in support of the hypothesized role of cognitive load in diminishing threat responses proposes that treatment of fear and anxiety related disorders could be understood as a reorganization of resources between the central-executive control network and the salience network^15^. Cognitively demanding tasks potentially induce such a reorganization by activating the central-executive control network and reducing activation in the amygdala, a key structure of the salience network^15^. Via this reorganization, cognitive demand can reduce threat-related responses^16,17^ that do not tax working memory, as well as conscious subjective feelings of fear^9,10^.

In the case of enhancing extinction learning, it could work in the following manner. During extinction, the amygdala is inhibited by the ventromedial prefrontal cortex (vmPFC), leading to a reduction in the expression of threat responses^18^. Interestingly, a decrease in blood oxygenation level-dependent (BOLD) signal, measured with functional Magnetic Resonance Imaging (fMRI) is observed in the amygdala during the execution of goal-directed eye movements^4^. It could therefore be the case that an additional inhibition of the amygdala during extinction, via an eye movement intervention, can strengthen extinction. Interestingly, amygdala inhibition is also found when participants perform a working memory task^4,19,20^ or play a computer game of Tetris^21^. Furthermore, greater decreases in BOLD-signal in the amygdala are observed when the cognitive load increases^15^. The reason the amygdala is inhibited may be due to a reciprocally coupling with the dorsal frontoparietal network shifting a distribution in resources to a cognitively-demanding task and away from threat^15^. What is unclear, however, is whether [1] other cognitively-demanding tasks that inhibit the amygdala can enhance extinction learning, and [2] whether the effectiveness of such an intervention can be enhanced by increasing the cognitive load.

If cognitive load is the therapeutic mechanism underlying the eye movement intervention, then any task that is cognitively demanding may potentially be a suitable intervention to enhance extinction learning. However, an ideal clinical intervention should allow for the cognitive load to be systematically increased to accommodate individual differences in cognitive capacity. A working memory task, such as an n-back task, might fulfil this requirement since the cognitive load can be systematically increased and performance can be assessed. In sum, we hypothesize that extinction learning can be enhanced when a working memory task is performed during extinction learning, and that the impact of a working memory task on extinction learning is greater when cognitive load is increased.

To test our hypothesis, participants completed an established Pavlovian threat conditioning/extinction/recall paradigm across two consecutive days with a 24 h interval. The first day comprised an acquisition session and an extinction session. The second day comprised a recall session to test for spontaneous recovery and recovery following reinstatement which allows us to test for the effects of the working memory intervention on recall under two different conditions. Conditioned stimuli (CS) were two images of a snake of which one (CS+) but not the other (CS−) was partially reinforced with an electrical shock (37.5% reinforcement rate). We tested the following predictions. First, we predicted that a working memory task would reduce the conditioned responses (i.e., skin conductance responses to the CS+ versus CS−) during extinction learning in a load-dependent fashion (Control group > Low-Load group> High-Load group) rather than any load (Control group > Low-Load group = High-Load group). Second, this load-dependent reduction in the conditioned response would persist 24 h later during recovery. Third, we predicted that individual differences in performance on the working memory task would be associated with the reduction in the conditioned response.

## Results

### Working memory performance embedded during extinction

**Figure 1:**
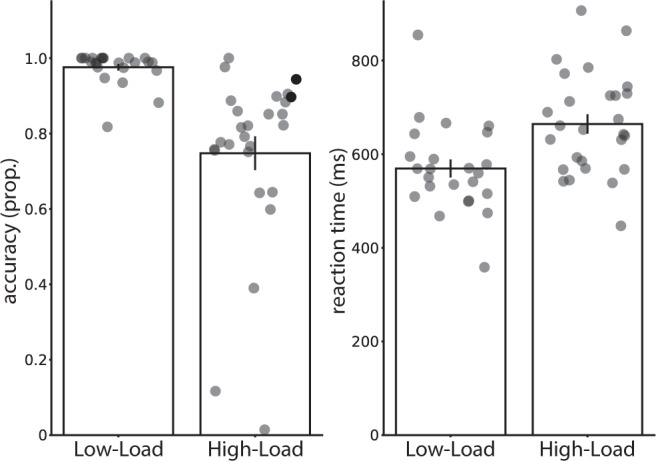
Average accuracy (proportion correct) and reaction times (ms) during the 1-back (Low-Load group) and 2-back (High-Load group) task embedded during extinction. Error bars represent ± standard error of the mean.

We first verified performance on the working memory intervention during extinction learning would be better in the Low-Load group compared to the High-Load group. Indeed, the Low-Load group performing a 1-back working memory task had higher accuracy [M = 98%, SD = 0.04%] than the High-Load group performing a 2-back working memory task [M = 75%, SD = 23%; t(49) = 4.70, p < 0.001, d = 1.32]. Furthermore, the Low-Load group [M = 569.31 ms SD = 94.96 ms] was faster than the High-Load group [M = 664.52 ms, SD = 107.33 ms; t(49) = −3.34, p = 0.002, d = −0.93] in responding to the targets. These results indicate a successful load-dependent working memory intervention. See Fig. 1.

### Skin conductance responses during acquisition

Next, we tested whether all groups showed a conditioned response during acquisition. We defined a conditioned response by a significant difference in skin conductance responses to the CS+ versus the CS−. Participants who did not show a numerically higher response to the CS+ compared to the CS− during acquisition were excluded from all analysis (see Participants of the Methods section for exclusion criteria).

First, we found a significant conditioned response across all trials and all groups [F(1, 72) = 113.66, p < 0.001, ${{\rm{\eta }}}_{{\rm{p}}}^{2}$= 0.61], which did not differ between the 3 groups (Control, Low-Load, High-Load) [F(2, 72) = 2.61, p = 0.081, ${{\rm{\eta }}}_{{\rm{p}}}^{2}$= 0.07]. Follow-up tests within each group separately showed that the Control group [t(23) = 5.96, p < 0.001, d = 1.22], the Low-Load group [t(23) = 6.55, p < 0.001, d = 1.34], and the High-Load group [t(26) = 6.68, p < 0.001, d = 1.29] all showed successful acquisition of conditioned threat.

**Figure 2:**
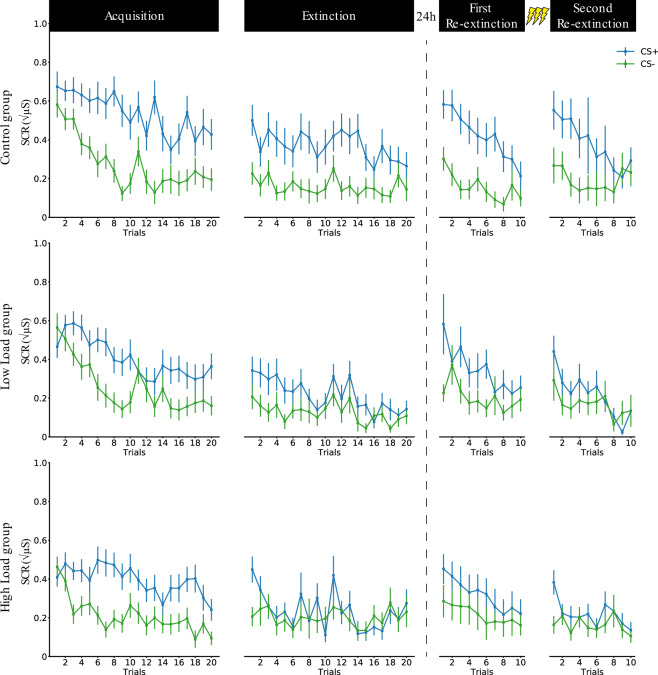
Time course of SCRs in response to the CS+ and the CS− across all trials an all phases for the Control group, the Low-Load group, and High-Load group separately plotted. CS = Conditioned stimulus; SCR = Skin conductance response. Error bars represent ± standard error of the mean.

**Figure 3:**
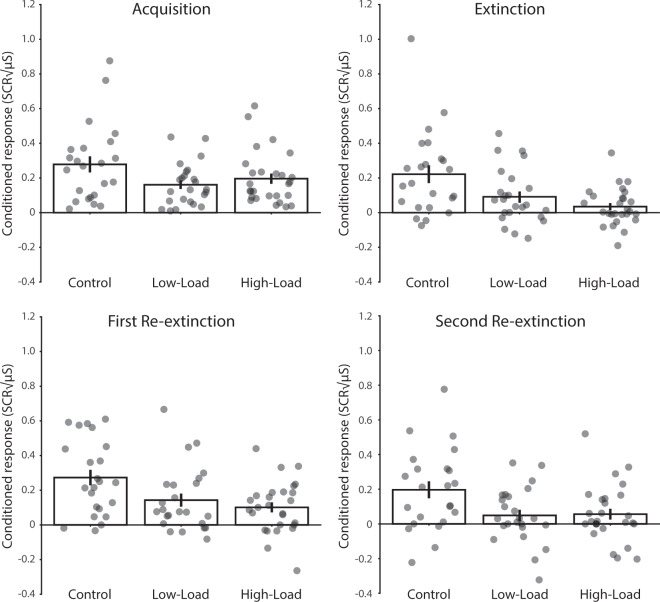
Average conditioned response (CS+ versus CS−) across all trials an all phases for the Control group, the Low-Load group, and High-Load group separately plotted. SCR = Skin conductance response. Error bars represent ± standard error of the mean.

Although not statistically significant, we observed a numerical difference in the magnitude of the conditioned response between the groups. Therefore, in addition to the results presented below, we performed a control analyses by adding the average conditioned response during acquisition as a covariate to all further analysis reported below. These additional analyses did not yield to any different results or conclusion, and we therefore report the results without this covariate. See Figs. 2 and 3.

### Skin conductance responses during extinction

Next, we investigated the effect of a working memory intervention during extinction learning in between CS presentations on the conditioned response during extinction learning. First, we found a significant conditioned response across all extinction trials and all groups [F(1, 72) = 30.92, p < 0.001, ${{\rm{\eta }}}_{{\rm{p}}}^{2}$= 0.30]. However, the strength in the conditioned response was significantly different between the 3 groups (Control, Low-Load, High-Load) [F(2, 72) = 6.74, p = 0.002, ${{\rm{\eta }}}_{{\rm{p}}}^{2}$= 0.16]. By directly comparing each group with each other we found that the conditioned response was stronger in the Control group compared to the Low-Load group F(1, 46) = 4.24, p = 0.045, ${{\rm{\eta }}}_{{\rm{p}}}^{2}$= 0.08] and the High-Load group [F(1, 49) = 12.07, p = 0.001, ${{\rm{\eta }}}_{{\rm{p}}}^{2}$= 0.20]. However, the conditioned response between the Low-Load and High-Load group [F(1, 49) = 2.28, p = 0.137, ${{\rm{\eta }}}_{{\rm{p}}}^{2}$= 0.04] did not significantly differ from each other during extinction.

We specifically hypothesized a load-dependent effect of the working memory intervention on the reduction in the conditioned response. We further investigated this by performing an additional analysis including planned, orthogonal, contrasts. In this analysis we aim to tease apart two predictions with two contrasts added to the same linear model. The first contrast assumes a load-dependent effect in which we expect a stepwise reduction in the conditioned response. The prediction is that the Control group would show the greatest conditioned response, the Low-Load group a smaller conditioned response, and the High-Load group the smallest conditioned response. The second contrast assumes that the presence of any load would reduce the conditioned response. Here the prediction is that the Low-Load group and High-Load group would show a reduction in the conditioned response compared to the Control group. This analysis indicated that both a linear reduction [t(72)=−3.619, p < 0.001] as well as the presence of any load [t(72)=−3.448, p < 0.001] explained the reduction in the conditioned response. Thus, these results suggest that cognitive load is enough to reduce the conditioned response and that the higher the cognitive load the stronger the reduction in the conditioned response.

Finally, the Control group [t(23) = 3.83, p < 0.001, d = 0.78] and Low-Load group [t(23) = 2.25, p = 0.035, d = 0.46] still exhibited a conditioned response during the last half of extinction, which was not the case for the High-Load group [t(26) = 0.42, p = 0.678, d = 0.08]. However, on the last trial of extinction there was no significant conditioned response for any of the groups [Control group: t(23) = 1.58, p = 0.128, d = 0.32, Low-Load group: t(23) = 0.69, p = 0.497, d = 0.14, and High-Load group: t(26) = 0.45, p = 0.655, d = 0.09]. Therefore, all groups showed evidence of extinction learning by the end of the session. See Figs. 2 and 3.

### Skin conductance responses during spontaneous recovery

The following day 24 h later, participants underwent a first re-extinction session to test for spontaneous recovery. When we looked at the entire time course of the first re-extinction session we found evidence of spontaneous recovery across all re-extinction trials and all groups [F(1, 69) = 63.30, p < 0.001, ${{\rm{\eta }}}_{{\rm{p}}}^{2}$= 0.48]. However, the magnitude in the conditioned response across the entire time course of the first re-extinction session was significantly different between the 3 groups (Control, Low-Load, High-Load) [F(2, 69) = 5.09, p = 0.009, ${{\rm{\eta }}}_{{\rm{p}}}^{2}$= 0.13]. By directly comparing each group with each other we found that the conditioned response was stronger in the Control group compared to the Low-Load group [F(1, 44) = 4.08, p = 0.0495, ${{\rm{\eta }}}_{{\rm{p}}}^{2}$= 0.08] and High-Load group [F(1, 47) = 9.61, p = 0.003, ${{\rm{\eta }}}_{{\rm{p}}}^{2}$= 0.17]. The conditioned responses did not significantly differ between the Low-Load and High-Load group [F(1, 47) = 0.89, p = 0.352, ${{\rm{\eta }}}_{{\rm{p}}}^{2}$= 0.02]. Again, the analysis including planned contrasts indicated that both the step-wise reduction [t(72)=− 3.172, p = 0.002] and the presence of cognitive load [t(72)=− 3.133, p = 0.002] explained the reduction in the conditioned response during spontaneous recovery induced by the working memory intervention. Additionally, we calculated the spontaneous recovery index (SRI), a typical index used to test for spontaneous recovery by subtracting the last trial of extinction from the first trial of re-extinction^4,22^. We did not observe spontaneous recovery on this index within the Control group [t(23) = 1.59, p = 0.125, d = 0.33] and there was no significant difference in SRI between groups [F(2, 69) = 0.44, p = 0.644, ${{\rm{\eta }}}_{{\rm{p}}}^{2}$= 0.01]. See Figs. 2 and 3.

### Skin conductance responses during recovery following reinstatement

On the same day, participants also underwent a second re-extinction session after reinstatement (i.e., after receiving 3 unsignaled UCSs). When we looked at the entire time course of the second re-extinction session we again found evidence of recovery across all trials during re-extinction and all groups [F(1, 69) = 21.16, p < 0.001, ${{\rm{\eta }}}_{{\rm{p}}}^{2}$= 0.23]. However, the strength in the conditioned response across the entire time course of the second re-extinction session was significantly different between the 3 groups (Control, Low-Load, High-Load) [F(2, 69) = 4.20, p = 0.019, ${{\rm{\eta }}}_{{\rm{p}}}^{2}$= 0.11]. We again directly compared each group with each other and found that the magnitude in the conditioned response was stronger in the Control group compared to the Low-Load group [F(1, 44) = 5.54, p = 0.023, ${{\rm{\eta }}}_{{\rm{p}}}^{2}$= 0.11] and High-Load group [F(1, 47) = 5.57, p = 0.023, ${{\rm{\eta }}}_{{\rm{p}}}^{2}$= 0.11]. The conditioned response did not differ significantly between the Low-Load and High-Load group [F(1, 47) <0.00, p = 0.989, ${{\rm{\eta }}}_{{\rm{p}}}^{2}$<0.01]. The planned contrast analysis again indicated both the step-wise reduction [t(72)=−2.515, p = 0.014] and the presence of cognitive load [t(72)=−2.927, p = 0.004] explained the reduction in the conditioned response during spontaneous recovery induced by the working memory intervention. Additionally, we calculated the recovery following reinstatement index (RRI) by subtracting the last trial of the first re-extinction session from the first trial of the second re-extinction session^4,22^ and did not observe a significant difference in RRI between groups [F(2, 69) = 0.06, p = 0.940, ${{\rm{\eta }}}_{{\rm{p}}}^{2}$<0.01]. See Figs. 2 and 3.

### Individual differences in working memory performance associated with a reduction in the conditioned response

**Figure 4:**
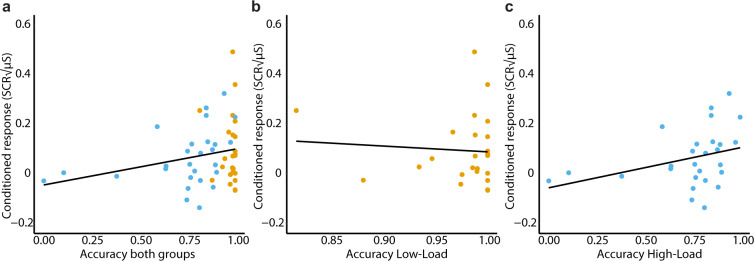
Performance (i.e., proportion accuracy) on the working memory intervention and average conditioned response across extinction, spontaneous recovery, and recovery following reinstatement. (**A**) Both groups plotted together for visualization purposes. (**B**) Accuracy scores in the Low-Load group performing the 1-back task did not predicted a reduction in the conditioned response [ρ(22) = −0.06,p = 0.78]. (**C**) Accuracy scores in the High-Load group performing the 2-back task predicted a reduction in the conditioned response [ρ (25) = 0.45,p = 0.019].

Finally, we investigated whether performance during the working memory intervention was associated with the reduction in the conditioned response during extinction learning, spontaneous recovery, and recovery following reinstatement. We averaged across all these sessions and performed a correlation test with n-back performance. Since the accuracy scores within each group (i.e., Low-Load and High-Load) was not normally distributed [1-back: W = 0.61, p = 7.953e-07, 2-back: W = 0.76, p = 2.618e-05], we performed a Spearman rank order correlation. Within the Low-Load group, we did not find a correlation between accuracy and the conditioned response [ρ(22)=−0.06, p = 0.78]. However, the performance within this group was very high and 42% of the participants performed with a 100% accuracy suggesting a possible ceiling effect with not enough variation to detect a relationship. Within the High-Load group, however, we observed a positive correlation between accuracy and the conditioned response [ρ(25)= 0.45, p = 0.019]: the higher the accuracy score, the stronger the conditioned response. We did not observe a relationship between reaction times and conditioned responses in the Low-Load group [r(22)= 0.05,p = 0.81], nor the High-Load group [r(25)=−0.29, p = 0.14]. This finding suggests that participants that exhibited higher performance (i.e., for those the task was less difficult to execute) on the 2-back task show less of a reduction in the conditioned response across the extinction and re-extinction sessions. See Fig. 4.

## Discussion

This study aimed to test the hypothesis that a working memory intervention embedded during extinction can enhance extinction learning, and that the impact of such an intervention is load-dependent. First, we found that the working memory intervention immediately reduced the conditioned response during extinction learning. Critically, we found that the reduction in the conditioned response remained 24 h later during spontaneous recovery, when no working memory task was performed, as well as for recovery after a reinstatement procedure. This suggests that a working memory intervention not only transiently reduces responses to threat but also enhances extinction learning. Next, we found that a reduction in the conditioned response was not only due to the presence of any cognitive load, but that stronger cognitive load had a bigger impact on the reduction in the conditioned response. We furthermore found that participants with lower performance on the 2-back working memory task (i.e., those for which the task was more difficult to execute) had a stronger reduction in the conditioned response. Together these findings suggest that, in addition to goal-directed eye movements^4^, a working memory intervention is able to enhance extinction learning, and that the benefits of a working memory intervention on extinction learning is dependent on the cognitive load.

Our finding of a reduction in immediate expression of the conditioned response during extinction is in line with earlier studies showing an immediate benefit of a cognitively-demanding task on attenuating subjective and physiological responses of arousal. For example, one study showed a reduction in the conditioned response when participants underwent a threat conditioning task while simultaneously executing a working memory task compared to when a group that did not perform a working memory task^16^. Another study found a reduction in fear-potentiated startle responses while simultaneously performing a working memory task^17^. Other studies have shown an immediate reduction in subjective affect ratings when threatening images where followed by the execution of a math task^23^. Moreover, emotionality and vividness of autobiographical memories, as well as intrusive memories, are reduced when memory reactivation is paired with working memory tasks^24–26^. Lastly, other types of cognitive control, such as emotion regulation, alter emotionality during autobiographical memory recollection (Denkova *et al*., 2015). We expand these findings by showing a working memory intervention can reduce sympathetic arousal in response to a previously learned threat.

Perhaps more interesting, we found that the reduction in the conditioned response during extinction persisted 24 h later when tested during spontaneous recovery (*i.e*., when no working memory task was being executed) and even following a reinstatement test. How can a working memory intervention have a beneficial effect on extinction learning? There are several mechanisms that may underlie the benefit of a working memory intervention on extinction learning

A first possibility is that, similar to an eye-movement intervention^4^, the working memory intervention reduces amygdala activation^4,15,19,20^, and via this reduction strengthens extinction learning. During extinction, the amygdala is inhibited by the ventromedial prefrontal cortex (vmPFC), leading to a reduction in the expression of threat responses^18^. It could therefore be the case that an additional inhibition of the amygdala during extinction, via cognitive load, could have strengthened extinction. Indeed, the amount of amygdala inhibition induced by a working memory task was found to be dependent on the cognitive load^15^. In line with this notion we have found evidence that when the cognitive load was higher the reduction in the conditioned response was stronger.

A second possibility is that the working memory intervention can have beneficial effects via novelty-facilitated extinction^27,28^. It was previously found that extinction can be enhanced by replacing a threat with a novel outcome, such as a non-aversive tone, during extinction learning^27,28^. One hypothesis outlining how novelty-facilitated extinction may enhance extinction is that the novel outcome reduces uncertainty during extinction. During standard extinction the US is omitted and the CS now either predicts a threat or the absence of a threat resulting in uncertainty. By replacing the threat with a novel outcome this uncertainty is reduced. It is therefore possible that a working memory intervention also reduces uncertainty since in our design the CS is always followed by a working memory task in the Low-Load and High-Load groups.

A third possibility is that the working memory intervention led to a devaluation of the US. In one study it was found that when participants were instructed to imagine the US and simultaneously made goal-directed eye movements, this led to a reduction in the conditioned response to the CS during the retention test^29^. However, in contrast to the previous study, in our design the US was not manipulated. Nevertheless, given that the CS is associated with the US, it is possible that replacing the US with a working memory intervention during extinction led to a reduction in the value of the US, and thereby a reduction in the conditioned response to the CS.

Finally, stress-related hormones are known to play a crucial role in strengthening learning and consolidation^30^, including extinction learning^31,32^. It has also been shown that a working memory task increases pupil dilation responses^33,34^ an index of sympathetic arousal. Furthermore, cognitively demanding tasks (i.e., mental arithmetic) are often used as a stress-induction procedure to increase cortisol^35,36^. Consistent with the hypothesis that stress hormones may modulate the consolidation of extinction/exposure learning, symptoms of fear and anxiety were shown to be reduced when glucocorticoids are administered before^37^ and after^38^ exposure therapy. It is possible that a working memory intervention, through an increase in stress-related hormones, strengthened the consolidation of extinction learning. However, somewhat inconsistent with this hypothesis is our observation of decreased sympathetic arousal during extinction learning as indicated by the immediate reduction in the conditioned response.

In the present study, we found that the working memory intervention reduced conditioned responses across the entire time course of extinction and re-extinction. We did not observe a between-group difference on the spontaneous recovery index (SSI) and the reinstatement recovery index (RRI). This is in contrast to previous studies^4,22^ that have used these measures to assess the effect of a manipulation on the recovery of threat responses. What could explain these differences? First, we found that our manipulation affected the entire time course of extinction learning. Second, we did not observe recovery on these indices within the Control group. In contrast to these previous studies, our design used snake images as CSs instead of geometrical shapes. Such prepared stimuli may have led to resistance to extinction^39^, which was specifically prominent in the Control group, which made it difficult to observe the recovery of the threat response on these standard indices. In our current study, the working memory intervention affected defensive responses across the entire time course of extinction and re-extinction, and the effect was not limited to a few trials.

In our design as well as a previous study^4^, the cognitively demanding intervention was performed following the CS presentation. Future studies should investigate whether performing a cognitively demanding task simultaneously with the CS presentation has similar consequences. Previous studies have found that when a cognitively demanding task is performed during threat learning, the conditioned response is reduced^16^, but it remains unclear what the consequences are with regards to extinction learning. This is a relevant question since it could shed new light on boundary conditions of EMDR treatment in which patients are instructed to keep the aversive memory in mind while performing goal-directed eye movements.

In sum, our results suggest that cognitive load induced by a working memory intervention embedded during extinction learning reduces transient threat responses as well as enhances extinction learning. We propose that not only goal-directed eye movements that are part of EMDR^40^ can enhance extinction learning^4^ but any task that is cognitively demanding may potentially be a suitable intervention to enhance extinction learning. We moreover found that a working memory intervention can enhance extinction in a load-dependent fashion. Since an ideal clinical intervention should allow for the cognitive load to be systematically increased to accommodate individual differences in cognitive capacity, a working memory task may potentially be more suitable as an intervention embedded within a clinical setting.

## Methods

### Participants

102 healthy volunteers (61 females, 41 males; 18–45 years [M = 24.4, SD = 6.6]) completed the entire study across two consecutive days. One additional participant was excluded due to a technical failure. Exclusion criteria for participation were as follows: current treatment or treatment in the last year of psychiatric, neurological, or endocrine disease, current treatment with any medication, average use of >3 alcoholic beverages daily, average use of recreational drugs, habitual smoking, uncorrected vision.

In total 29 participants were excluded after completion of the study based on one or more of the following criteria: 1) a smaller response than 0.05 μS in response to the unconditioned stimulus (UCS; n = 13), 2) a mean CS+ responses being numerically smaller than a mean CS− response during the acquisition phase (n = 13), or 3) a lack of contingency awareness (n = 11). The final sample consisted of 75 participants across three groups (Control group, n = 24, Low-Load group, n = 24, High-Load group, n = 27). We excluded “non-responders” and “non-learners” since our study is aimed at investigating extinction learning^27,41–43^ for which successful acquisition is crucial.

Lastly, since the stimuli in the experimental design included images of snakes, participants scoring above 23 on the Snake Phobic Questionnaire (SNAQ) were not eligible to participated. This would ensure participants were not phobic of these stimuli. No individual, however, scored above this value. All participants provided written informed consent approved by the University Committee on Activities Involving Human Subjects at New York University (Institutional Review Board #2016-2) and the study was conducted in accordance with these guidelines and regulations. Participants received payment ($20 per hour) for their participation.

### Experimental design

**Figure 5:**
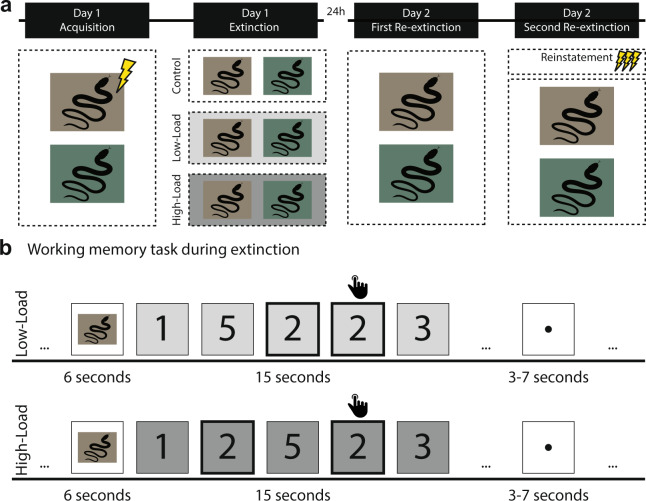
Overview of the experimental design. (**A**) The experiment took place on two consecutive days and included an acquisition session (day one), an extinction session (day one), a first re-extinction session to test for spontaneous recovery, and a second re-extinction session to test for recovery following reinstatement (day two). The conditioned stimuli (CS) consisted of two images of snakes obtained from the freely available International Affective Picture System (IAPS) database (https://csea.phhp.ufl.edu/media/iapsmessage.html). An illustration of these images are displayed in the figure. (**B**) During the 1-back (Low-Load) working memory group participants were instructed to press a button when the current digit had appeared one positions back in the sequence. For the 2-back (High-Load) working memory group participants were instructed to press a button when the current digit had appeared one positions back in the sequence.

Participants were tested in a differential delay threat conditioning paradigm^4,22,44^ on two consecutive days with a 24 h interval in a between subject-design (Control, Low-Load, High-Load). The first day comprised an acquisition session and an extinction session and the second day a recall session. The stimulus set across the two days consisted of two images of snakes taken from the International Affective Picture System IAPS; ^45^; number 1026 and 1090 as conditioned stimuli (CS). On day one during acquisition, one cue (CS+, 6 s duration) was partially reinforced (37.5% reinforcement rate) with a mild electrical shock to the right wrist (*i.e*., the UCS). The other cue (CS−, 6 s duration) was never reinforced. In total, there were 52 trials of which 20 were CS+ unreinforced, 12 CS+ reinforced, and 20 CS trials. The CS+ s reinforced, CS+ s unreinforced, and CS−s were presented in a pseudorandom order. The ISI was jittered between 18 s and 22 s with an average of 20 s. Extinction included 40 CS trials (20 trials per CS, 6 s duration). For the control group each CS was followed by a jittered ISI between 18 s and 22 s with an average of 20 s. In the Low-Load working-memory group each CS was followed by a 1-back working memory task with a duration of 15 s and followed by a jittered ISI between 3 s and 7 s with an average of 5 s. In the High-Load working-memory group each CS was followed by a 2-back working memory task with a duration of 15 s and followed by a jittered ISI between 3 s and 7 s with an average of 5 s. On day two, the experiment started with a first re-extinction session, which included 21 CS trials. The first presentation was always a CS− and discarded from the analyses, the remaining trials consisted of 10 CS+ and 10 CS− trials (6 s duration). After this session there was a reinstatement procedure^46^ consisting of 3 un-signaled UCS presentations (ISI: 10 s). Following this, participants underwent a second re-extinction session, which included again first a CS− trial which was discarded from the analysis and the remaining trials consisted of 10 CS+ trials and 1- CS− trials (6 s duration). During both phases the ISI was jittered between 18 s and 22 s with an average of 20 s. The experiment was created and presented using the Python library *Expyriment* version 0.9.1b2^47^ implemented in Python 2.7. See Fig. 5 for an overview.

### Working memory intervention

During each of the 40 trials (15 s duration) during extinction the Low-Load and High-Load working memory group saw a random sequence consisting of 10 single digits. Each digit was presented for 1100 ms, followed by an interstimulus interval (ISI) of 400 ms. Participants in the Low-Load working-memory group were asked to detect whether the current item had appeared one positions back in the sequence and the High-Load working-memory group were asked to detect whether the current item had appeared two positions back in the sequence. Participants were instructed to make a button press when detecting such a target. One out of four digits was a target.

### Peripheral stimulation

Electrical shocks were delivered via two Ag/AgCl electrodes (EL503, Biopac) and conductance gel (GEL100, Biopac) attached to wrist of the right arm using a SD9 Square Pulse Stimulator (Grass Technologies) device. Shock duration was 200 ms which co-terminated with the CS presentation. During a standardized shock intensity adjustment procedure, each participant received and subjectively rated several shocks between 0 and 9, allowing shock intensity to converge to a level experienced as uncomfortable, but not painful (M = 6.71, SD = 1.10). Participants received between 5 and 8 shocks during this procedure between ending up with a level between 15 V and 55 V (M = 35.77, SD: 10.09 V). The level was set on day 1 and remained the same on day 2 for the reinstatement procedure.

### Peripheral measurements

Electrodermal activity was assessed using two Ag/AgCl electrodes (EL507, Biopac) and NaCl gel (GEL101, Biopac) attached to the left palm. Skin conductance responses (SCR) were automatically scored with additional manual supervision using Autonomate^48^ implemented in Matlab 2017b (MathWorks). We opted to use the magnitude method, since it has been considered the standard method of scoring SCRs^49^. SCR amplitudes (measured in μSiem) were determined for each trial within an onset latency window between 0.5 and 6.5 s after stimulus onset, with a minimum rise time of 0.5 s and a maximum rise time of 6 s after response onset. In case of multiple responses fulfilling these requirements, the largest response was counted. Reinforced trials were omitted, and all other response amplitudes were square-root transformed prior to statistical analysis and corrected for individual responses to the UCS (i.e., shock) on each day.

The first two trials of acquisition were always a CS− and a CS+ (order was randomized) and were discarded from the analysis. The first trial of the first re-extinction and second re-extinction session was always a CS− and discarded from the analysis. Four repeated-measures ANOVAs were conducted with CS (CS+, CS−) and Time (Early, Late) as a within-subject factors. Group (Control, Low-Load, High-Load) was included as a between-subject factor. For the re-extinction sessions the presentation order (CS+ or CS−) of the second and third trial was included as a between-subject factor. We ran an additional linear model including only two planned, orthogonal, contrasts to specifically test the hypothesis working memory intervention would reduce the conditioned response in a load-dependent fashion rather than a load-independent fashion. The first contrast assumed a load-dependent effect in which we expected a stepwise reduction in the conditioned response were the Control group would show the greatest conditioned response, the Low-Load group a smaller conditioned response, and the High-Load group the smallest conditioned response. The second contrast assumed that the presence of any load would reduce the conditioned response were the Low-Load group and High-Load group would show a reduction in the conditioned response compared to the Control group. To test for spontaneous recovery, the conditioned response (CS+ versus CS−) on the last trial of extinction was subtracted from the conditioned response on the first trial during the first re-extinction session^4^. The reinstatement recovery index was calculated in a similar way by subtracting the conditioned response on the last trial during the first re-extinction session from the conditioned response on the first trial during the second re-extinction session^4^. For these analyses, the presentation order (CS+ or CS−) of the second and third trial was included as a between-subject factor.

### Statistical testing

Statistical analysis were performed using R^50^. Analysis of variance (ANOVA) was performed using the ez package the planned contrast analysis was performed using the lm function in the stats package. Partial eta squared (Pη²) or Cohen’s d effect size estimates are reported for all relevant tests. Shapiro-Wilk Normality Test was used to test for normality. Spearman rank order correlations were used for correlations across participants. Alpha was set at 0.05 throughout and two-tailed t-tests were conducted.

## Data Availability

Data and code are available on Open Science Framework: https://osf.io/r25f4/
