# Publisher Correction: A cognitively demanding working-memory intervention enhances extinction

**DOI:** 10.1038/s41598-020-69587-7

**Published:** 2020-07-29

**Authors:** Lycia D. de Voogd, Elizabeth A. Phelps

**Affiliations:** 1 Department of Psychology, New York University, New York, NY 10003 USA; 2 Department of Psychology, Harvard University, Cambridge, MA 02138 USA

Correction to: *Scientific Reports* https://doi.org/10.1038/s41598-020-63811-0, published online 27 April 2020

The original version of this Article contained an error in the title of the paper, where the word “Research Article” was incorrectly added. This has now been corrected in the PDF and HTML versions of the Article.

